# Using High Molecular Precision to Study Enzymatically Induced Disassembly of Polymeric Nanocarriers: Direct Enzymatic Activation or Equilibrium-Based Degradation?

**DOI:** 10.1021/acs.macromol.0c02263

**Published:** 2021-01-26

**Authors:** Gadi Slor, Roey J. Amir

**Affiliations:** † School of Chemistry, Faculty of Exact Sciences, Tel-Aviv University, Tel-Aviv 6997801, Israel; ‡ Tel Aviv University Center for Nanoscience and Nanotechnology, Tel-Aviv University, Tel-Aviv 6997801, Israel; § Blavatnik Center for Drug Discovery, Tel-Aviv University, Tel-Aviv 6997801, Israel; ∥ ADAMA Center for Novel Delivery Systems in Crop Protection, Tel-Aviv University, Tel-Aviv 6997801, Israel; ⊥ The Center For Physics And Chemistry Of Living Systems, Tel-Aviv University, Tel-Aviv 6997801, Israel

## Abstract

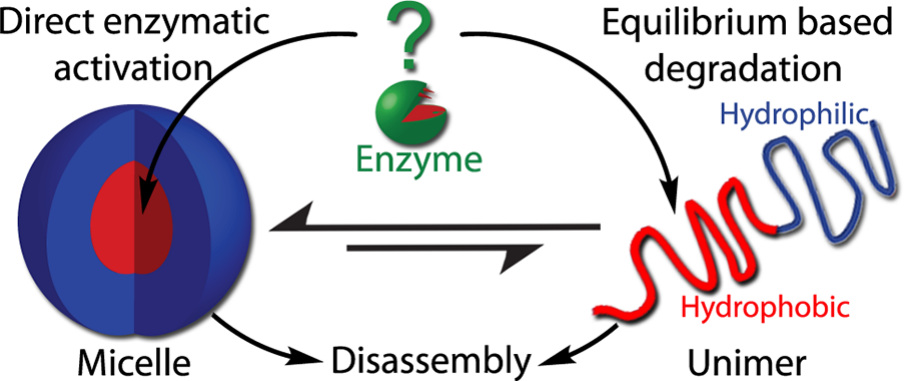

Enzyme-responsive polymers and their assemblies offer great potential to serve as key materials for the design of drug delivery systems and other biomedical applications. However, the utilization of enzymes to trigger the disassembly of polymeric amphiphiles, such as micelles, also suffers from the limited accessibility of the enzyme to moieties that are hidden inside the assembled structures. In this Perspective, we will discuss examples for the utilization of high molecular precision that dendritic structures offer to study the enzymatic degradation of polymeric amphiphiles with high resolution. Up to date, several different amphiphilic systems based on dendritic blocks have all shown that small changes in the hydrophobicity and amphiphilicity strongly affected the degree and rate of enzymatic degradation. The ability to observe the huge effects due to relatively small variations in the molecular structure of polymers can explain the limited enzymatic degradation that is often observed for many reported polymeric assemblies. The observed trends imply that the enzymes cannot reach the hydrophobic core of the micelles, and instead, they gain access to the amphiphiles by the unimer–micelle equilibrium, making the unimer exchange rate a key parameter in tuning the enzymatic degradation rate. Several approaches that are aimed at overcoming the stability–responsiveness challenge are discussed as they open the way to the design of stable and yet enzymatically responsive polymeric nanocarriers.

## Introduction

Polymeric micelles of various dimensions and compositions have been widely explored as potential drug carriers for hydrophobic drug molecules. It is clear that for a micelle to serve as a drug delivery platform it must be extremely stable against dilution and degradation before it reaches its target. At the same time, a drug release mechanism is needed to allow the delivery of the drug to the target site. Many release mechanisms have been explored, ranging from simple diffusion of the drugs from the carrier to more sophisticated stimuli-responsive polymeric micelles^1−5^ that can release the drug on demand in response to specific stimuli. These include changes in temperature^6−11^ and pH,^12−18^ irradiation with UV–vis light,^19−25^ or the presence of analytes such as thiols.^26−29^ Among the various types of stimuli, enzymatic activation or degradation may offer great potential due to the overexpression of specific enzymes in different diseases.^30−35^ For example, enzymes such as matrix metalloproteinases^36^ or cathepsin B,^37^ which are overexpressed in various types of cancer, can potentially be utilized to trigger the release of chemotherapeutic drugs selectively at the site of the tumor.

Together with the great potential of enzymes to trigger the release of hydrophobic drugs, enzymatic activation holds one key difference from other types of stimuli—the accessibility of the enzyme to the enzyme-responsive moieties. In comparison with dimensionless stimuli such as light and temperature, or low molecular weight species such as protons (or hydronium ions) in the case of pH-responsive assemblies, the significantly larger dimensions of enzymes can drastically limit their accessibility to the responsive groups.

In the past few years, we have been studying polymeric amphiphiles based on linear PEG as the hydrophilic block and dendron containing enzymatically cleavable lipophilic end-groups as the hydrophobic block.^38−41^ Taking advantage of the high molecular precision that emerges from using a dendron as the hydrophobic block, we studied how fine-tuning of the amphiphilicity, mostly by altering the structure of the hydrophobic dendron, affects the self-assembly and enzymatic degradation of our PEG-dendron amphiphiles. In this Perspective we will share our understanding of the enzymatic degradation mechanism by discussing our results together with key examples of enzyme-responsive polymeric assemblies that have inspired our own research. We will focus only on highly precise dendritic systems and their utilization to study the effect of fine structural changes on the enzymatic activation/degradation and cargo release with high resolution. In our opinion, the study of such systems, which benefit from the well-defined and precise dendron-based structure of the amphiphile, can shed more light on the limitations and challenges that need to be addressed to gain a better understanding of the design principles of enzymatically degradable polymeric nanocarriers and enzyme-responsive materials in general.

## Limited Enzymatic Degradation of Polymeric Assemblies

Striking evidence for the limited accessibility of an enzyme to its substrates when they are attached to a polymeric backbone was reported by the Hawker group in 2009.^42^ In that work, monofunctional PEG was used to polymerize protected phosphate bearing styrenic monomers, which after the deprotection of the phosphate group yielded a double hydrophilic block copolymer. The polymers were designed to be soluble in aqueous solution and to self-assemble upon enzymatic cleavage of the phosphate side groups from the styrenic block as its hydrophobicity increased due to the removal of the charged solubilizing moieties. This enzymatic activation transformed the diblock copolymers to become amphiphilic, leading to their self-assembly into spherical colloidal nanostructures. Interestingly, when following the degree of dephosphorylation directly by ^31^P NMR, a substantial amount of phosphate groups (∼40% for the shorter block with degree of polymerization (DP) of 13 and ∼10% for a longer block with DP of 30) remained on the styrenic block. Further evidence for the residual phosphate groups was revealed in a fluorescent assay using pyrene, which indicated greater polarity of the enzymatically assembled structures in comparison with the fully dephosphorylated amphiphilic block copolymer, which was used as a control. These results demonstrate that once the polymers become amphiphilic and start to self-assemble, the residual phosphate groups, which are linked to the core-forming block, become inaccessible to the enzyme, and their cleavage rate becomes negligible. Similar trends in enzymatic degradation of phosphorylated polypeptides due to limited accessibility of the enzyme to the phosphate side groups were recently reported by Gupta and co-workers.^43^

**Figure 1:**
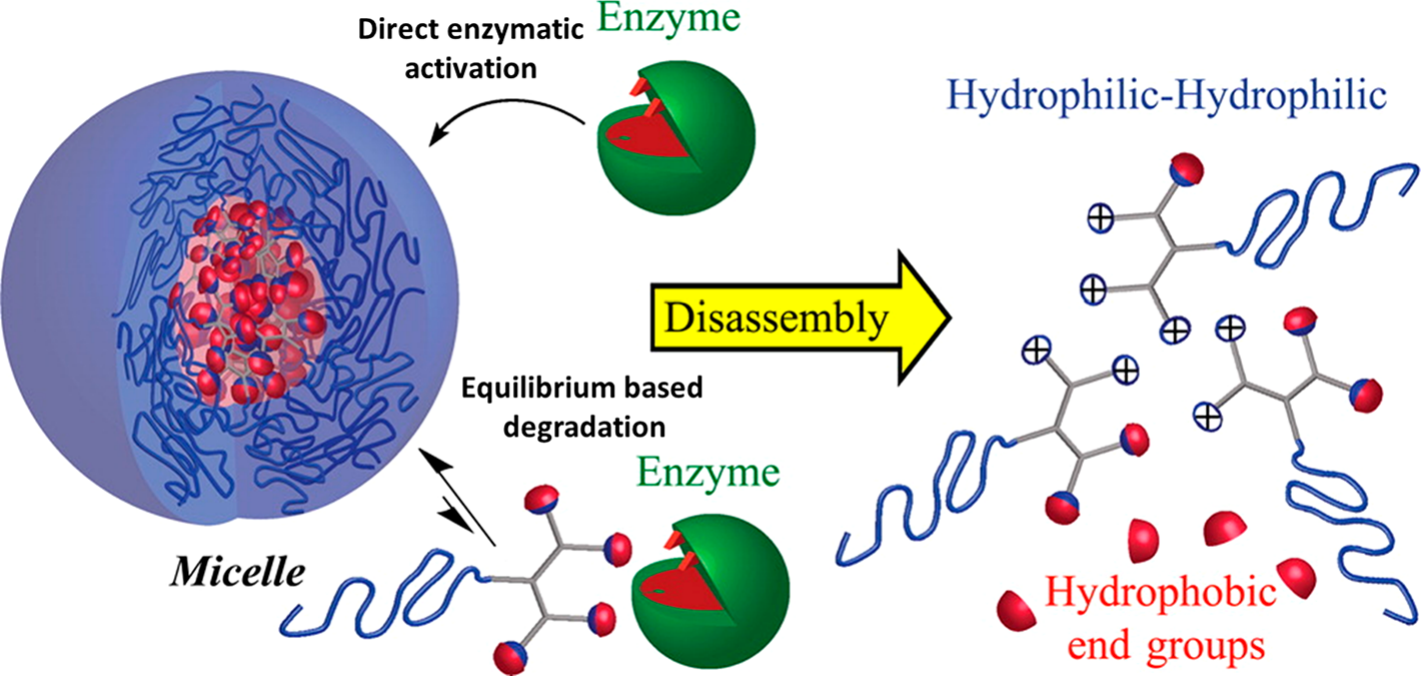
Schematic representation of two hypothetical enzymatic activation pathways: direct enzymatic activation in which enzyme penetrates through the micellar shell or equilibrium-based degradation where the enzyme cleaves the hydrophobic end-groups of the of the amphiphiles in their unimer state. Reproduced with permission from ref (38).

On the basis of these results, one could argue that the design of polymeric assemblies that contain enzymatically cleavable lipophilic groups is not realistic as the activating enzyme will not be able to reach the hydrophobic core, which is expected to accommodate the cleavable groups. This limited accessibility was also demonstrated for polymeric assemblies with enzyme-cleavable shells as reported by the group of Heise for amphiphilic polymers containing hydrophilic peptides as the hydrophilic block and polystyrene (PS) or poly(*n*-butyl acrylate) as the hydrophobic block.^34^ In their paper, Heise and co-workers hypothesized that the limited degradation of the amphiphiles based on PS was due to the higher *T*_g_ of the PS chains and the resulting limited exchange and escape from the micelles. The high stability of self-assembled DNA-based nanoparticles, as reported by the Mirkin group,^44^ further supports the limited access of degrading enzymes even to the outer shell of nanosized assemblies. However, reading through the literature, it is clear that there are also numerous papers and reviews that describe the enzymatically induced disassembly of different polymeric amphiphiles.^45−51^ Do these reports stand in contradiction to the hypothesis of limited access of the enzyme to the polymeric chains, which result in higher stability and poor responsiveness of polymeric assemblies toward enzymatic degradation? Or is there another mechanism that can still allow access of the enzyme to its hydrophobic substrates (Figure 1)?

## Using High Moleuclar Precision to Study the Enzymatic Degradation of the Hydrophobic Block

One of the key contributors to the research of enzymatically degradable polymeric nanocarriers is the group of Prof. Thayumanavan, which over the years introduced very elegant molecular designs that were based on Janus-type dendritic branching units bearing both hydrophilic and hydrophobic moieties. This unique design offers high molecular precision, which emerges from using dendritic amphiphiles, while the enzyme-responsiveness of the polymeric amphiphiles is achieved by linking the hydrophobic moieties by an enzymatically cleavable ester bond. These dendritic branching units have been utilized to prepare dendritic amphiphiles of various generations and degree of polymerization, which were found to self-assemble into aggregates with diameters of 100–200 nm, as measured by dynamic light scattering (DLS). Enzymatic cleavage of the hydrophobic moieties by porcine liver esterase (PLE) should result in increased hydrophilicity of the degraded dendrons, leading to their disassembly.

**Figure 2:**
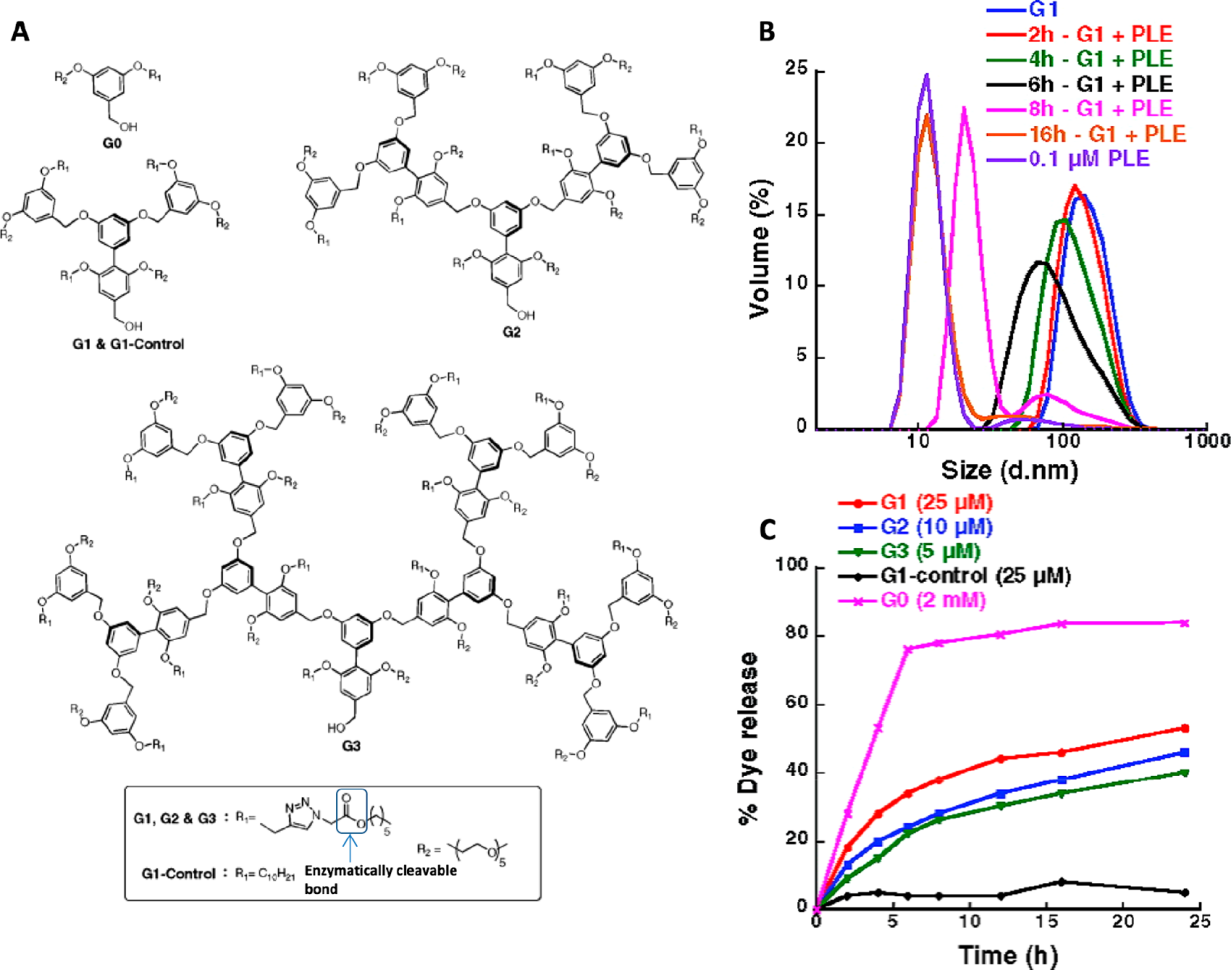
(A) Structures of generation 0–3 enzyme-responsive dendritic amphiphiles and (B) their micellar aggregates disassembly upon exposure to PLE as monitored by DLS and (C) release of encapsulated pyrene. Reproduced with permission from ref (52).

When looking at one of the first papers of the Thayumanavan group in this field,^52^ a clear trend could be observed from dye release experiments—a zero-generation (G0) dendron bearing a single hydrophilic chain and a single enzymatically cleavable aliphatic ester dissembled much faster than higher molecular weight dendrons (first- to third-generation dendrons), which showed significantly slower dye release and disassembly rates as the generation of the dendrons increased (Figure 2). The observed trend of the enzymatic response rate was attributed to the greater steric protection of the higher-generation dendrons, which can limit the accessibility of the enzyme to its substrate.

**Figure 3:**
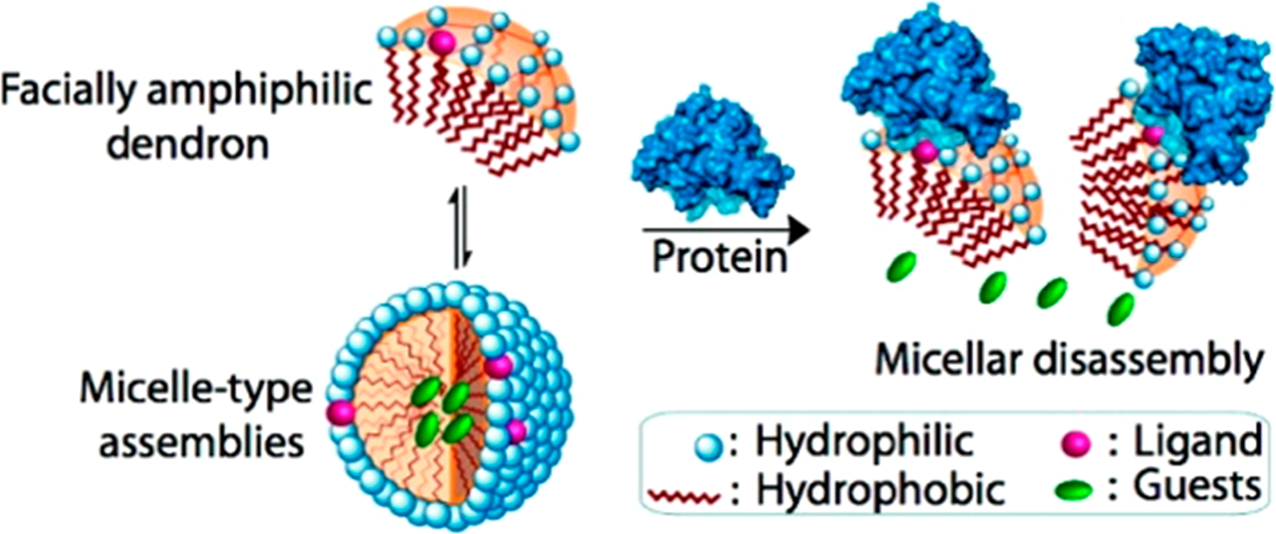
Schematic of protein–ligand binding-induced disassembly of dendritic micellar assemblies and resultant guest release. Reproduced with permission from ref (53).

In parallel to their reports on enzyme-triggered disassembly of dendrimer-based assemblies, the Thayumanavan group also studied the ability of protein–ligand interaction to induce disassembly (Figure 3). Dendrons of different generations were precisely decorated with single ligands such as biotin or dinitrophenyl (DNP), which could interact with extravidin^53^ or anti-DNP immunoglobulin G antibody (IgG),^54^ respectively. These studies included also the impressive capability to precisely label the focal, middle layer, or periphery of first- and second-generation dendrons with biotin and the use of molecular dynamics to gain a deeper insight into the structural parameters that govern the protein–ligand interactions.^55^ The results showed that the interaction of the dendrons with the protein, through ligand–protein binding, significantly change the amphiphilicity of the dendron–protein complexes leading to disassembly of the micellar assemblies. On the basis of the trends in disassembly and cargo release, it seems that the protein-responsive amphiphiles interact with the protein at their unimer state.^32,54^ This hypothesis was a key finding that was later suggested also for enzyme-responsive amphiphiles, which just like their protein-responsive analogues, need be accessible to the enzyme to allow the enzymatic reaction to take place.

**Figure 4:**
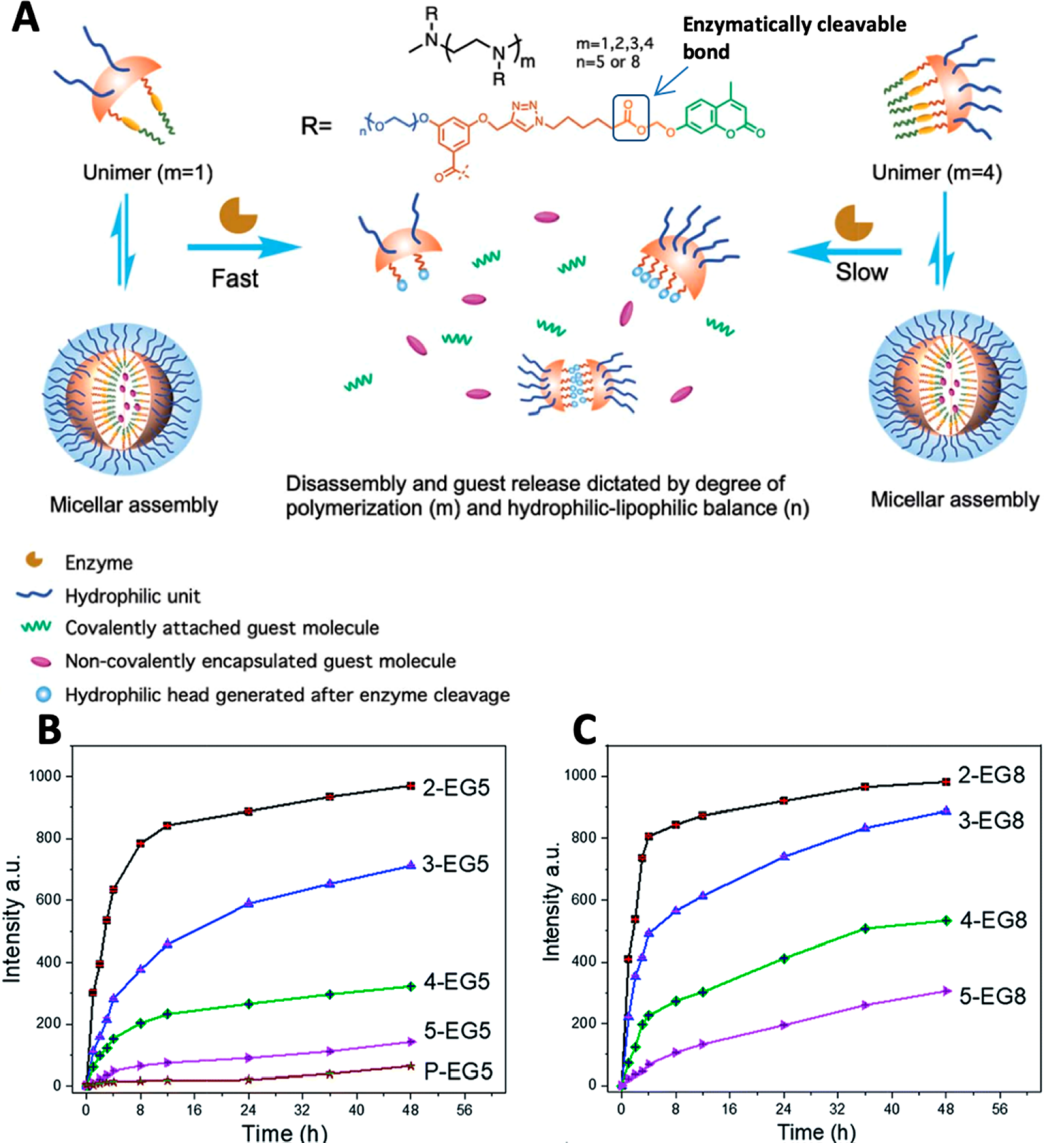
(A) Schematic representation of enzyme-induced disassembly and guest release from oligomeric assemblies and enzymatic hydrolysis of oligomeric assemblies based on coumarin release in oligomers with (B) pentaethylene glycol and (C) octaethylene glycol chains as hydrophilic moiety. Reproduced with permission from ref (56). Copyright 2019 The Royal Society of Chemistry.

In a recent paper, looking deeper into the effect of the molecular weight of the amphiphiles on the enzymatically induced disassembly, the Thayumanavan group designed G0 dendrons bearing penta- or octaethylene glycol chains as the hydrophilic moieties and a 4-methylumbelliferone dye linked through an enzymatically cleavable acetal–ester bond.^56^ Conjugating these dendrons to well-defined oligoethylene imine with specific numbers of repeat units (2–5) yielded oligomeric amphiphiles with distinct molecular weights (Figure 4A). The high molecular precision of this methodology allowed them to prepare amphiphiles with increasing molecular weights that had the exact same hydrophilic-to-hydrophobic ratio, as this is derived from the dendritic unit. Taking advantage of the increase in fluorescence of the released dyes upon enzymatic cleavage of the ester bonds and the subsequent hydrolysis of the hemiacetal group, the fluorescence emission was used to evaluate the enzymatic degradation rates (Figure 4B and C). In addition to the release of the coumarin moieties, the release of encapsulated hydrophobic dye was also monitored to follow the enzymatic induced disassembly of the polymeric assemblies. Upon comparison of the different oligomers, the results clearly showed slower degradation rates for the oligomers with more repeat units or for those with shorter oligoethylene glycol chains. While the effect of the length of the oligoethylene glycol chains can be clearly contributed to the changes in the hydrophilic to hydrophobic ratio, the effect of the degree of polymerization illustrates the greater contribution of the hydrophobic segments to the stability of the self-assembled structures toward enzymatic degradation. This was attributed to the change in the dynamics of the unimer–assembly equilibrium, which seems to be the key parameter that governs the rate and degree of enzymatically induced disassembly.

To shed more light on the importance of the unimer–assembly equilibrium, Thayumanavan and his group utilized photo-crosslinking of a coumarin-containing amphiphile, aiming to decrease the ability of unimers to escape the polymeric assembly and get cleaved by the activating enzyme.^57^ The results clearly showed a significant reduction in the rates of release of both bound and encapsulated dyes, indicating that the enzymatic activation indeed takes place at the unimer level. However, as crosslinking may also limit the ability of the enzyme to penetrate into the assembled structure, the less probable option of entry of the enzyme directly into the core of the polymeric assembly cannot be completely disproved.

As mentioned in the Introduction, in the past seven years our group used a different dendron-based molecular design to study the enzymatic activation of polymeric amphiphiles. To gain high molecular precision, we decided to take advantage of the unique molecular architecture of PEG-dendrons that was pioneered by Frechet, Gitsov, Hawker, and Wooley^58,59^ in the early 1990s and later utilized by many researchers including the groups of Fréchet,^60^ Hawker,^61^ Aida,^62^ Gitsov,^63^ Malkoch,^64^ Kakar,^65^ and others as thoroughly reviewed by Gillies and co-workers.^66^ Our design was based on amphiphilic hybrids of a linear PEG as the hydrophilic block and a dendron with enzymatically cleavable lipophilic end-groups as the hydrophobic block. By developing a step efficient methodology for accelerated synthesis of the dendron from the PEG by combining orthogonal amidation and thiol–yne/ene reactions, we could fine-tune the degree of amphiphilicity of the amphiphiles and study their self-assembly and enzymatically induced disassembly.

**Figure 5:**
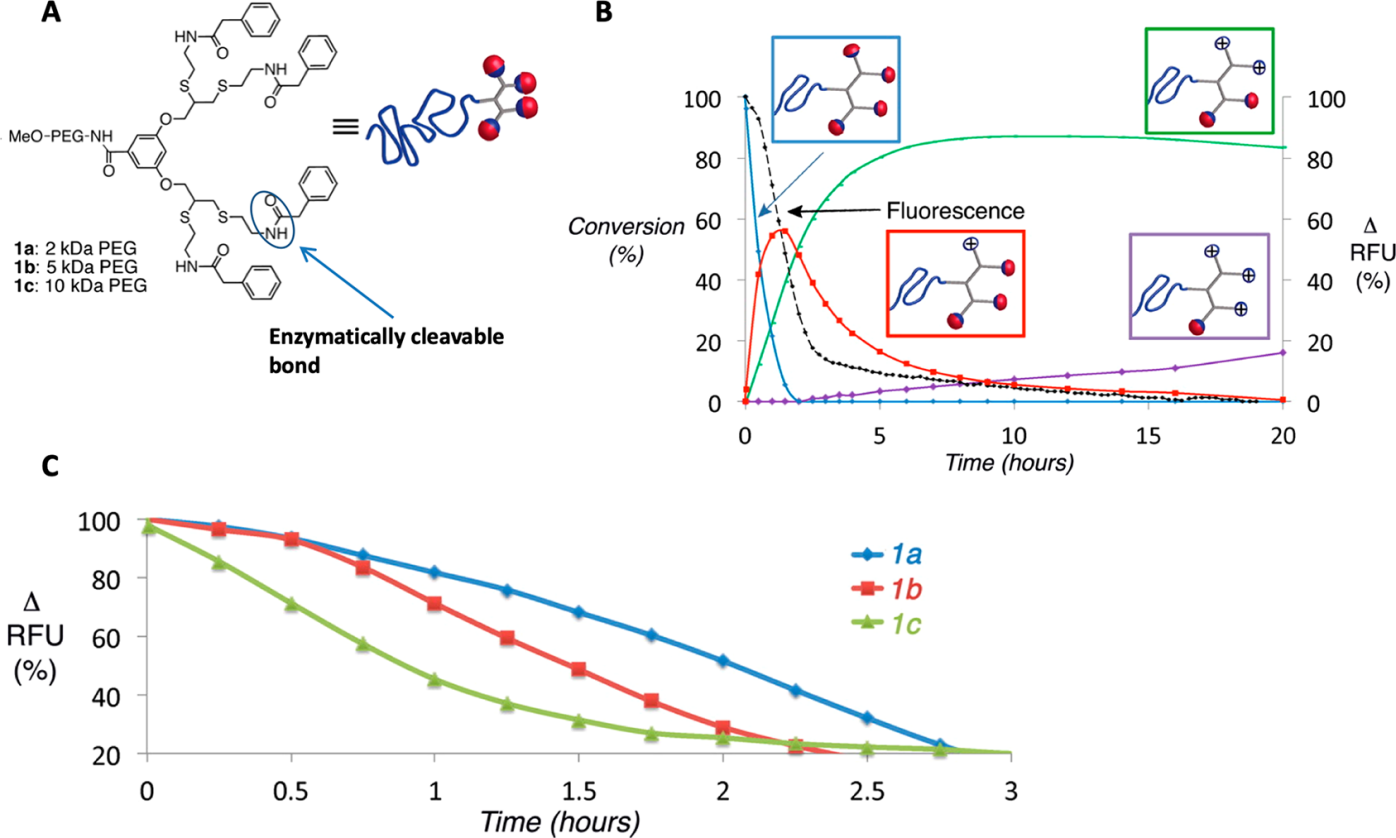
(A) Chemical structure of PEG-dendron hybrids with four penicillin G amidase cleavable end-groups and different PEG molecular weights. (B) Change in Nile red fluorescence intensity and HPLC analysis of the enzymatic degradation of the PEG-dendron hybrid **1b**. Partially degraded intermediates are shown schematically. (C) Comparison of the disassembly rates (fluorescence assay) of micelles formed by PEG-dendron hybrids **1a**–**c**. Reproduced with permission from ref (38).

In our first paper on enzymatic disassembly of polymeric micelles, we prepared three diblock amphiphiles bearing amidase-cleavable dendrons and PEG chains of different molecular weights: 2, 5, and 10 kDa (Figure 5A).^38^ All amphiphiles self-assembled into nanosized micelles with increasing diameters (from 11 to 18 nm) as the PEG chains got longer. In addition, we found that the increase in length of the PEG chain resulted also in higher critical micelle concentration (CMC) values (7, 12, and 22 μM for the 2, 5, and 10 kDa PEG-based amphiphiles, respectively). The utilization of a dendron to present the cleavable end-groups allowed all of the cleavable end-groups to be terminal and highly symmetrical, which was found to be highly advantageous for the detailed characterization of the enzymatic degradation. Furthermore, the monodispersity of the dendritic block allowed us to use HPLC to directly follow the cleavage of the end-groups by the activating enzyme and track the formation of both partially and fully cleaved amphiphiles (Figure 5B). Combining HPLC, DLS, and florescence spectroscopy (Figure 5C), we could show that the amphiphiles with longer PEG chains and higher CMC values also showed faster disassembly. The direct correlation between the higher CMC values and faster degradation of the parent amphiphiles and disassembly rates provided a strong indication that the enzymatic activation occurs in the free unimer state as its concentration can be expected to be reflected by the higher CMC values, similarly to the reports of the Thayumanavan group.

Although the enzymatic degradation was not completed and partially cleaved intermediates were accumulating, by comparing the HPLC and fluorescence results, we could show that a single cleavage was sufficient to cause the disassembly of the micelles (Figure 5B) as was also confirmed by DLS. Interestingly, we noticed that the first enzymatic cleavage of the parent hybrids was faster for the amphiphiles with the longer PEG blocks. However, once there were no more micelles present in the solution, it seemed as if the PEG chain became a steric barrier that led to slower degradation of the monocleaved intermediates as their concentrations reached 45, 60, and 65 mol % for the 2, 5, and 10 kDa PEG-based amphiphiles, respectively.^38^ These results are in good agreement with a very recent paper by our group, looking at the reverse role of the architecture of the PEG chain as amphiphiles based on a V-shaped PEG chain, which showed faster disassembly but also slower complete enzymatic degradation in comparison with the analogues amphiphiles composed of linear PEG with the same molecular weight.^67^

**Figure 6:**
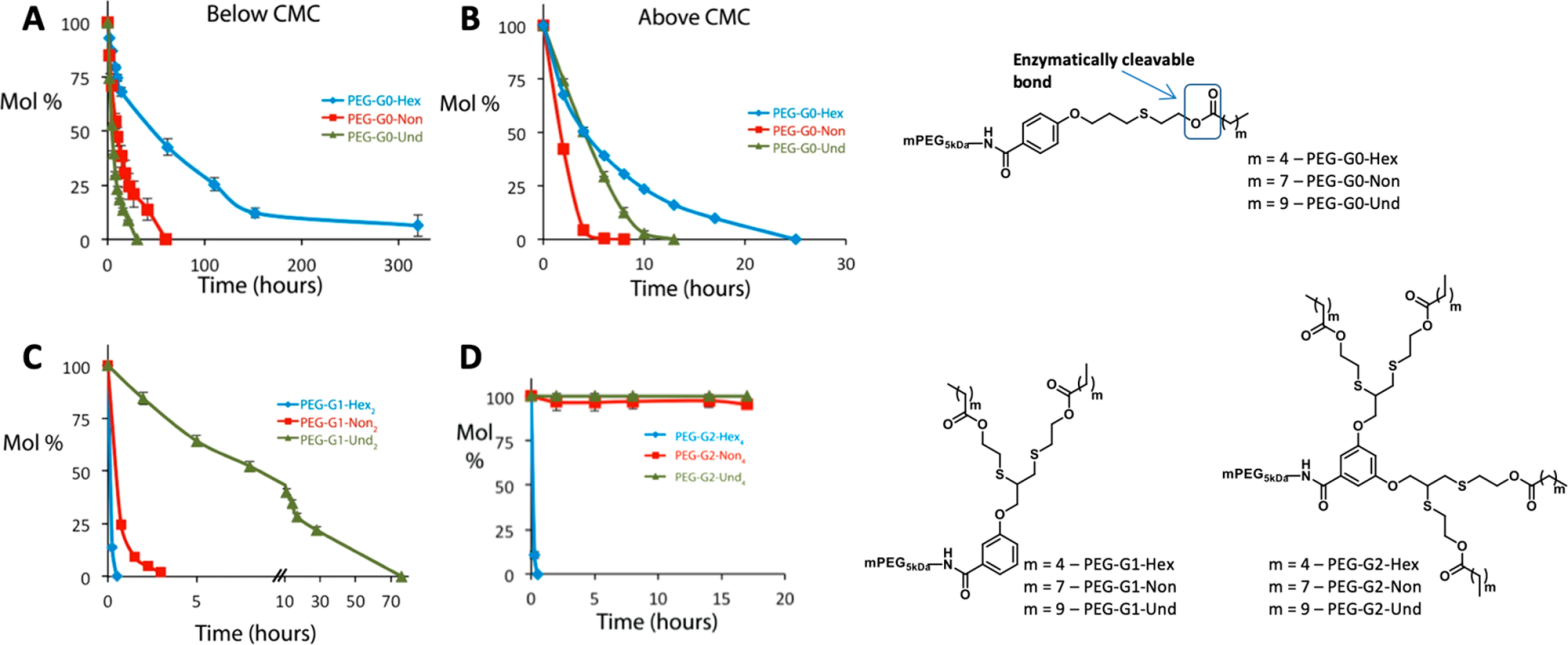
Enzymatic degradation kinetics and chemical structures of PEG-GO below (A: 10 μM) and above (B: 600 μM) its CMC, PEG-G1 (C: 100 μM), and PEG-G2 (D: 25 μM). Reproduced with permission from ref (40).

Following our observation of the correlation between the increase in CMCs for amphiphiles with larger hydrophilic block and their faster enzymatically induced disassembly, which indicated an equilibrium-based enzymatic activation, we set to study the effect of changes in the hydrophobic block. Toward this goal, we designed PEG-dendron amphiphiles bearing enzymatically cleavable end-groups with different degrees of hydrophobicity. Using our accelerated synthetic methodology, we prepared amphiphiles with four hexanoate, nonanoate, or undecanoate end-groups that can be cleaved by an esterase (Figure 6).^40^ The changes in hydrophobicity led to relatively small changes in CMC values (2–4 μM). However, the size of the micelles was strongly influenced by the increase in hydrophobicity as the diameter of the micelles increased by 8 nm going from the hexanoic-based amphiphiles to the nonanoic ones and another 8 nm increase for the undecanoate. This increase in size cannot be rationalized when considering only the longer length of the end-groups, which got elongated by only a few methylene units and hence can contribute to an increase of a few angstroms. Using small-angle X-ray scattering (SAXS), we could estimate that the aggregation number nearly doubled upon the increase in hydrophobicity of the end-groups, leading to the significant increase in diameter. Strikingly, the differences in the degradation and disassembly rates were even more significant, and while the hexanoate-containing amphiphiles were readily degraded upon addition of the enzyme, the nonanoate-containing PEG-dendrons were fully cleaved only after 24 h when incubated with significantly higher concentration of the enzyme (Figure 6D). The amphiphiles containing the most hydrophobic undecanoate end-groups were found to be highly stable, and nearly no degradation or disassembly was observed. These results could be attributed to the differences in the unimer–micelle equilibrium and the unimer exchange rate as expected from an equilibrium-based enzymatic activation. However, it could also be that the longer aliphatic chains are simply poorer substrates for the enzyme and hence get degraded much slower. To examine this, we prepared amphiphiles with a zero-generation dendrons, which had a single hydrophobic chain and hence were expected to have relatively high CMC values. The three amphiphiles were indeed found to have relatively high CMC values (∼60 μM for PEG-G0-Hex, ∼40 μM for PEG-G0-Non, and ∼20 μM for PEG-G0-Und), as expected. These high CMC values enabled us to use the HPLC and study their enzymatic degradation at a concentration of 10 μM, which is well below their CMC. This allowed us to directly estimate their suitability to serve as substrates for the activating esterase. It was fascinating to see that under these conditions when the amphiphiles should be present mostly as unimers, the polymers with the longer aliphatic chains degraded faster than the ones with the shorter chains. When we tested the same amphiphiles at a much higher concentration of 600 μM, which is well above their CMCs, the trend got mixed, and the degradation of the nonanoate-containing amphiphile was the fastest. These results provide strong support for the equilibrium-based mechanism, as if the enzyme could penetrate into the micelles, the longer undecanoate bearing amphiphiles should have remained the fastest to degrade.

It is very important to note that the observed kinetic trends and the reverse correlation between hydrophobicity of the polymeric amphiphiles and the responsiveness of their nanoassemblies have been observed not only for PLE but also for other enzymes such as penicillin G amidase.^38,39,68^ Furthermore, these trends were also reported for linear amphiphiles such as the elastase-responsive assemblies that were reported by Heise^34^ and the acid phosphatase-responsive polymers reported by Hawker.^42^ The similar behaviors for different enzymes and polymeric architectures demonstrate that indeed the deeper understanding that is obtained from using well-defined dendritic amphiphiles can be generic and applicable to other polymeric systems.

## Overcoming the Stability-Responsiveness Limitation

The dependence of the enzymatic degradation on the unimer–micelle equilibrium severely limits the ability to design stable enzyme-responsive assemblies. On the one hand, to make the assembly stable enough to withstand the high dilution and harsh conditions upon their introduction into the body, the hydrophobicity or overall molecular weight should be increased. On the other hand, the increased stability will result also in limiting the unimer–micelle equilibrium, leading to poorly or nondegradable polymeric assemblies. Hence, overcoming the reverse correlation between stability and enzyme-responsiveness is, in our opinion, the key challenge that needs to be addressed when designing novel enzyme-responsive polymeric assemblies.

**Figure 7:**
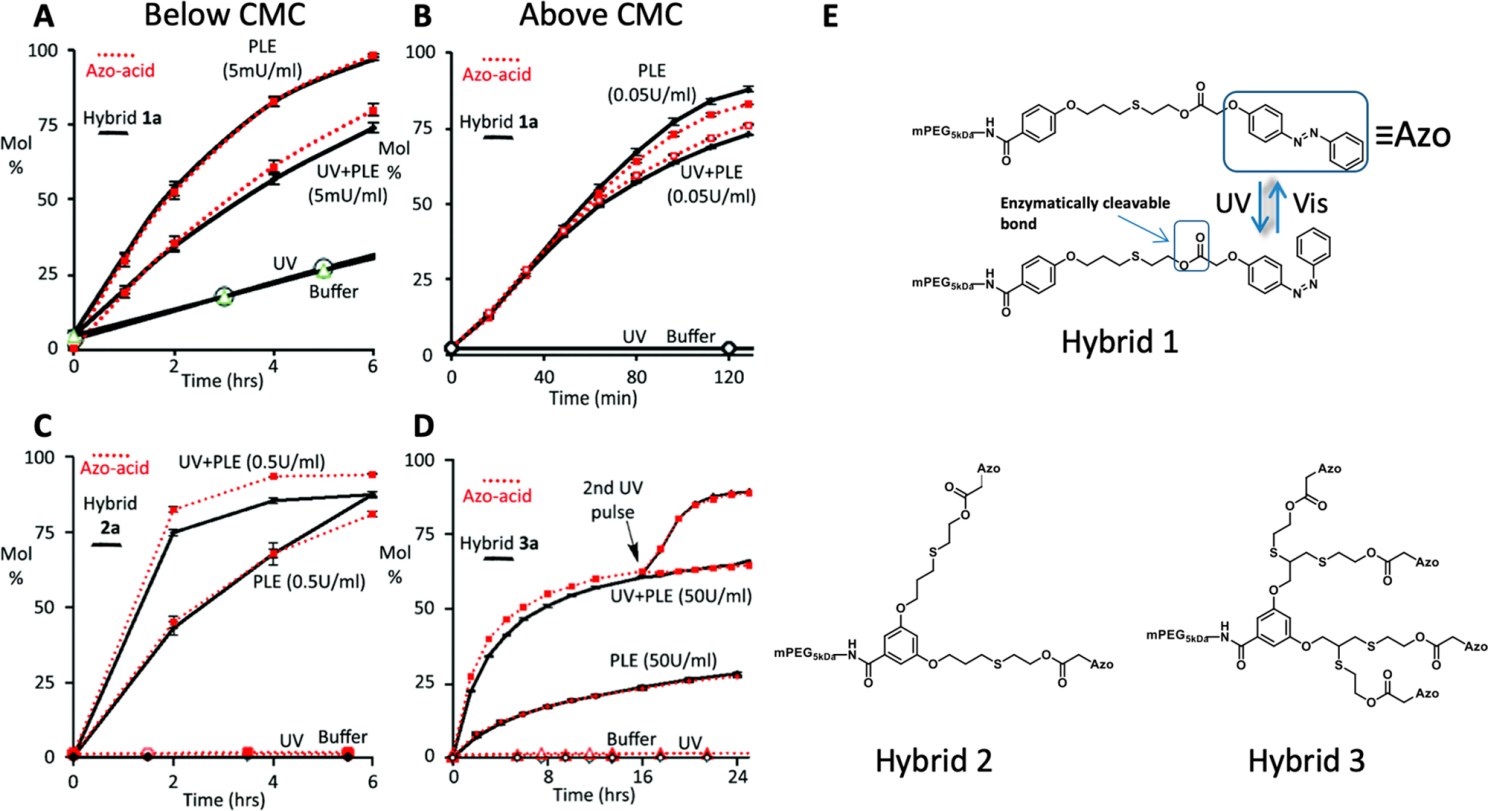
Kinetic analysis of the enzymatic hydrolysis to release azo end-groups (red dotted lines) and the alcohol-containing corresponding degraded polymer (solid black lines) under various conditions: UV pulse and PLE, PLE only, UV pulse and buffer. (A) Hybrid 1 below (20 μM) and (B) above (320 μM) its CMC value, (C) hybrid 2 (160 μM), and (D) hybrid 3 (80 μM). (E) Chemical structures of PEG-dendron hybrids containing one, two, and four enzymatically cleavable azo end-groups and demonstration of it cis/trans photoisomerization. Reproduced with permission from ref (69). Copyright 2016 Royal Society of Chemistry.

One possible solution is to combine additional stimuli-responsive moieties that allow tuning the amphiphilicity of the amphiphiles in addition to the enzymatic degradability of the hydrophobic block. An example for this approach was reported by Harnoy and Slor et al., who designed a dual responsive system that contained both photoresponsive azobenzene moieties and enzymatically degradable bonds (Figure 7).^69^ The reported design took advantage of the ability of azobenzene to switch from the more hydrophobic trans isomer to the more polar cis isomer upon UV irradiation, which has been widely utilized for controlling the polarity and function of low molecular weight switches,^70,71^ polymeric systems,^25,72−74^ and surface-modified metallic nanoparticles.^75^ The photoresponsive azobenzene groups were linked to the dendron through ester bonds, which can be hydrolyzed by an esterase, as enzyme-responsive groups. Three different amphiphiles containing zero- to second-generation dendrons were synthesized and used for studying the effect of photoisomerization on the enzymatic degradation and disassembly of the amphiphiles (Figure 7E). Once again, taking advantage of the high molecular precision of the dendritic block, HPLC was used to directly follow both the photoisomerization and enzymatic degradation. Interestingly, when measuring the enzymatic degradation of the zero-generation-based amphiphile, which had the smallest hydrophobic block, below its CMC, the trans-containing amphiphiles degraded faster than the cis-containing amphiphiles (Figure 7A). This indicated that the more hydrophobic trans-isomer is the better substrate for the enzymatic cleavage. However, when the zero-generation amphiphiles were tested at a concentration above their CMC, the degradation rates became similar (Figure 7B). The differences in degradation rates between the cis- and trans-containing amphiphiles became even more substantial for the first-generation (Figure 7C) and second-generation amphiphiles (Figure 7D), which showed a reverse trend as the cis-containing isomers were cleaved much faster. The enzymatic hydrolysis of the second-generation amphiphiles that had most of their end-groups in the trans form reached only 25% degradation after 24 h. On the other hand, the UV-irradiated samples that had most of the end-groups in the cis-form showed much faster degradation, reaching nearly 60% after 16 h and nearly 100% after a second UV irradiation (this was needed as the cis-isomer can thermally convert back to the more stable trans-isomer). The obtained results provided further support for the equilibrium-based mechanism as if the enzyme could penetrate into the micelles, one would expect that the amphiphiles containing the trans-isomers will degrade faster as was observed for the zero-generation amphiphiles below their CMC. The faster degradation of the cis-containing amphiphiles was attributed to the increase in polarity and the resulting faster unimer–micelle exchange. It is important to note that, unlike other reports in which the photoisomerization was sufficient to cause the disassembly (or at least deformation) of the polymeric assemblies, in the presented case, the isomerization, which was clearly observed by HPLC, was not significant enough to lead to the disassembly of the micelles.

**Figure 8:**
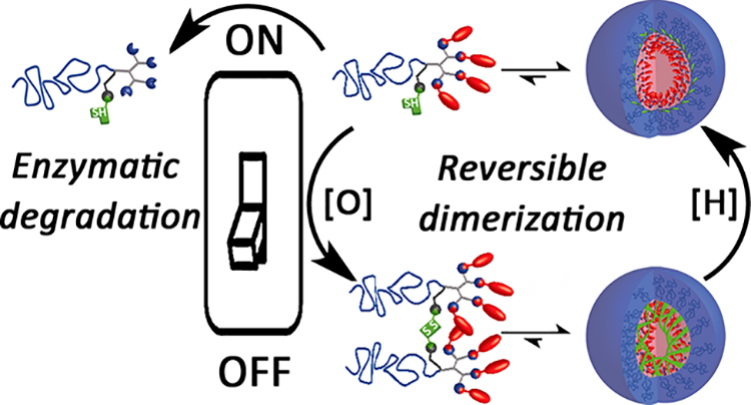
Schematic presentation of the reversible dimerization of polymeric amphiphiles as a switching mechanism between highly stable micelles (dimers) and micelles composed of reduced monomeric amphiphiles that can undergo enzymatically induced disassembly. Reproduced with permission from ref (76).

Another approach that utilized dual responsive amphiphiles was illustrated in a paper by Rosenbaum et al.^76^ In this report, PEG-dendron amphiphiles bearing a single thiol moiety were used to form dimers held by a disulfide bond. Strikingly, although the amphiphilicity of the dimeric amphiphiles was exactly the same as the monomeric amphiphiles, the dimeric ones were found to be extremely stable toward enzymatic degradation. This significant difference in stability can be clearly attributed to the increase in molecular weight, demonstrating again the greater contribution of the hydrophobic block to the stability of the self-assembled structure. When incubated with the enzyme in the presence of dithiothreitol as a reducing agent, the dimeric amphiphiles could break back to the monomeric form, and the enzymatic degradation and disassembly followed the same rates as obtained for a control structure that could not undergo dimerization. This approach (Figure 8) opens new directions for controlling the stability of the assembled structures as it takes advantage of the change in the molecular weight of the amphiphiles rather than the decrease in hydrophobicity as done in the azobenzene-based systems.^69,73^

**Figure 9:**
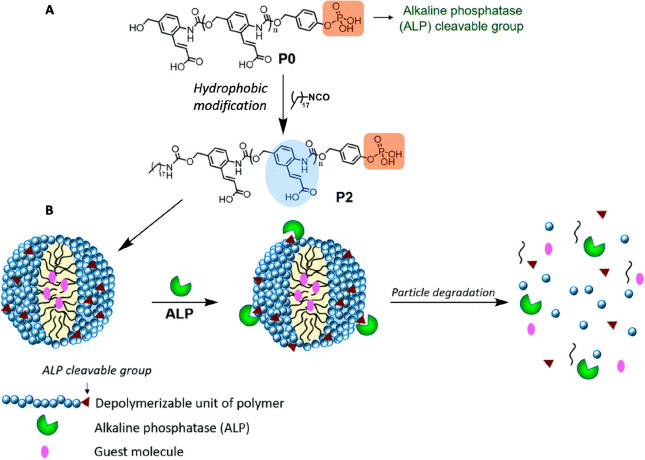
(A) Structure of the ALP triggerable polymer, P0, and its hydrophobic modification into P2. (B) Proposed schematic of particle formulation using P2 and its triggered disassembly in response to ALP. Reproduced with permission from ref (77). Copyright 2020 Royal Society of Chemistry.

Both above-described approaches of dual responsive amphiphiles are based on enhancing the enzymatic responsiveness of the amphiphiles by reducing their hydrophobicity or molecular weight and hence accelerating the unimer–micelle exchange, which is critical for the enzymatic activation. A very different approach was very recently reported by Thayumanavan and co-workers that utilized an elegant molecular design that allows the translation of an enzymatic cleavage on the surface of polymeric assemblies into complete degradation of the assembled structures (Figure 9).^77^ The design is based on the use of self-immolative polymer as the hydrophilic block. As in previous reports by Shabat,^78^ Moore,^79^ Gillies,^80^ and others,^81−83^ the Thayumanavan group took advantage of the ability of the self-immolative polymers to undergo controlled self-degradation upon cleavage of its head group. The polymers were functionalized with phosphate ester as head group and a long aliphatic chain as tail group and were shown to self-assemble into nanoparticles with diameters of ∼250 nm that could degrade by cleavage of the phosphate head group by alkaline phosphatase (ALP). The uniqueness of the system is the fast response that is achieved by the ability to present the hydrophilic enzyme-cleavable groups on the surface of the polymeric assemblies, which make them highly accessible to the enzyme. This elegant approach, which places the enzymatic cleavage sites on the surface of the polymeric assemblies and hence significantly enhances their availability to the activating enzyme, overcomes the need to balance between stability and responsiveness. However, it comes with two significant price tags: release of electrophilic species that are being formed during the self-degradation process and might have some toxicity and the need for custom synthesis using a relatively limited number of backbones.

## Conclusions and Future Perspectives

There is no doubt that enzyme-responsive polymers and their assemblies hold great potential as key materials for the design of smart drug delivery systems and other biomedical applications. However, using enzymes to trigger the disassembly of polymeric amphiphiles brings also a significant challenge due to the comparable dimensions of enzymes and micelles, which limit the accessibility of the enzyme. Taking advantage of the high molecular precision that dendritic structures offer, we can study the enzymatic degradation of polymeric amphiphiles with high resolution. Up to date, several different amphiphilic systems containing dendritic blocks have all shown that small changes in the hydrophobicity and amphiphilicity strongly affected the degree and rate of enzymatic degradation. In all the systems that were described above, increasing the degree hydrophobicity by altering the type or number of lipophilic side/end-groups led to rapid increase in stability against the enzymatic degradation. In addition, increasing the molecular weight of the amphiphiles, while keeping the hydrophilic-to-hydrophobic ratio constant, also led to a significant increase in stability.

The observed trends imply that the enzymes cannot reach the hydrophobic core of the micelles and have limited access also to the hydrophilic chains in the shell. Instead, amphiphiles can become significantly more accessible in their unimer form, making the unimer–micelle equilibrium and exchange rates key parameters in tuning the enzymatic degradation rate. Although the observed stability-responsiveness trends have been mostly studied for esterase (PLE)-responsive systems, similar behaviors were also reported for penicillin G amidase- and elastase-responsive amphiphiles, demonstrating that the unimer–assembly equilibrium-based activation is indeed general and not specific only to esterase. At the same time, it is clear that the repertoire of activating enzymes needs to be extended to include disease-associated enzymes and that these enzyme-responsive systems should be studied in more complex environments such as serum and blood to evaluate their performance under more relevant conditions to the final biomedical application of delivering drugs in a selective manner in the body.

The ability to observe how relatively small variations in either the molecular weight of the hydrophobic block or the hydrophilic/hydrophobic ratio of well-defined dendritic amphiphiles strongly affected their degradation, implies that for most types of polymers, small changes in their molecular weight due to their inherited polydispersity can result in a broad range of enzymatic responsiveness. Hence, polymers with a relatively small hydrophobic block will be readily degraded while polymers with a higher degree of hydrophobicity might degrade very slow or become nondegradable because of their greater thermodynamic and/or kinetic stabilities. This deeper understanding of the fine balance between stability and responsiveness of amphiphiles can explain the partial enzymatic degradation that is observed for many reported polymeric assemblies. The risk of poor degradability due to small variations in the degree of hydrophobicity can be extremely important in the field of polymer therapeutics when using hydrophilic polymers for preparing polymer–drug conjugates. These polymeric carriers are often prepared by the postpolymerization step of conjugating the lipophilic drugs to the side groups of the polymer. As the exact number of drugs per polymer chain cannot be precisely controlled beyond the average number of drugs per chain, these procedures will result in ensembles composed of polymers with different number of conjugated hydrophobic drugs. Such polymers are expected to self-assemble into micelles or other types of assemblies bearing the hydrophobic drugs in their core. The variation in the hydrophobicity may lead to rapid enzymatic degradation of the polymers carrying a smaller number of drugs, while polymers with a larger number of drugs might become poorly degradable or nondegradable because of their higher thermodynamic and kinetic stabilities. This wide range in responsiveness means that the more hydrophobic polymers, which carry more drug molecules and hence contain the major part of the loaded drugs, might not be able to release the conjugated drugs, leading to an overall poor drug release profile.

By highlighting the need to balance between the stability of the assemblies and their responsiveness to the enzyme, the reported studies of high molecular precision systems further support the need to overcome the stability-responsiveness barrier. To date, several approaches including multiresponsive systems and self-immolative amphiphiles capable of self-destructing upon cleavage of its end-groups have been developed. While there is no doubt that these approaches open the way toward highly stable and yet enzymatically responsive polymeric assemblies, further research and development are needed to address the synthetic challenges that are associated with these more complex platforms. Furthermore, in the next steps, the design of enzyme-responsive nanocarrier systems should take into account not only the types of stimuli in the biological environment but also the sequence at which biological cues are encountered by the drug delivery systems. Last but not least, while the high molecular precision of polymeric systems has not been translated into the clinic, there is no doubt that such systems provide a highly valuable and essential tool for studying the how small changes in the polymeric structures affects their function.

## Abbreviations

CMC: critical micelle concentration
DLS: dynamic light scattering
HPLC: high-pressure liquid chromatography.
